# Distinctive Properties and Powerful Neuromodulation of Na_v_1.6 Sodium Channels Regulates Neuronal Excitability

**DOI:** 10.3390/cells10071595

**Published:** 2021-06-25

**Authors:** Agnes Zybura, Andy Hudmon, Theodore R. Cummins

**Affiliations:** 1 Program in Medical Neuroscience, Paul and Carole Stark Neurosciences Research Institute, Indiana University School of Medicine, Indianapolis, IN 46202, USA; azybura@iu.edu; 2 Biology Department, School of Science, Indiana University-Purdue University Indianapolis, Indianapolis, IN 46202, USA; 3 Department of Medicinal Chemistry and Molecular Pharmacology, College of Pharmacy, Purdue University, West Lafayette, IN 47907, USA; ahudmon@purdue.edu

**Keywords:** voltage-gated sodium channel, action potential, axon initial segment, sodium currents, channelopathies, post-translational modifications, protein-protein interactions

## Abstract

Voltage-gated sodium channels (Navs) are critical determinants of cellular excitability. These ion channels exist as large heteromultimeric structures and their activity is tightly controlled. In neurons, the isoform Na_v_1.6 is highly enriched at the axon initial segment and nodes, making it critical for the initiation and propagation of neuronal impulses. Changes in Na_v_1.6 expression and function profoundly impact the input-output properties of neurons in normal and pathological conditions. While mutations in Na_v_1.6 may cause channel dysfunction, aberrant changes may also be the result of complex modes of regulation, including various protein-protein interactions and post-translational modifications, which can alter membrane excitability and neuronal firing properties. Despite decades of research, the complexities of Na_v_1.6 modulation in health and disease are still being determined. While some modulatory mechanisms have similar effects on other Nav isoforms, others are isoform-specific. Additionally, considerable progress has been made toward understanding how individual protein interactions and/or modifications affect Na_v_1.6 function. However, there is still more to be learned about how these different modes of modulation interact. Here, we examine the role of Na_v_1.6 in neuronal function and provide a thorough review of this channel’s complex regulatory mechanisms and how they may contribute to neuromodulation.

## 1. Introduction

A well-functioning and healthy brain is dependent on the ability of neurons to integrate and relay impulses. These impulses are mediated by the activity of voltage-gated sodium channels (Navs) by controlling the initiation and propagation of electrical signals, which are fine-tuned by myriad signaling events to contribute as critical regulators of neuronal excitability [1].

**Figure 1:**
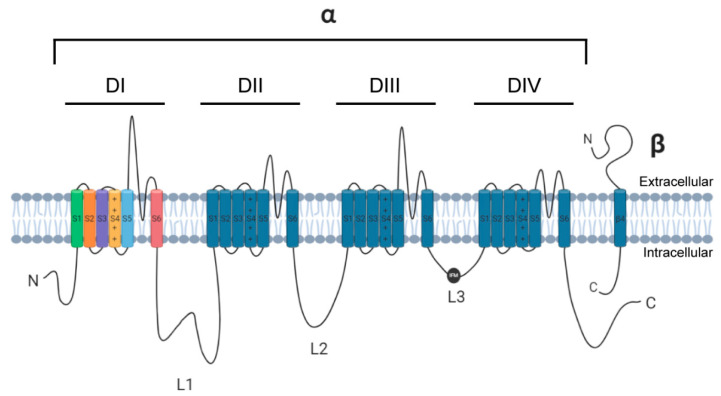
Linear schematic of a voltage-gated sodium channel α subunit and an auxiliary β subunit. L3 depicts the IFM motif (black circle) for channel fast inactivation.

Navs exist as large complex heteromultimeric structures consisting of a pore-forming α subunit that may be covalently or non-covalently bound to auxiliary subunits, chief among these being β subunits (β1–4) (Figure 1) [2,3,4]. The Nav α subunit is comprised of a ~2000-amino acid polypeptide chain folded into a complex tertiary structure organized into four homologous transmembrane domains (DI-DIV), each containing six α-helical segments (S1–S6). The S1–S4 segments comprise the voltage sensing domain (VSD) which contains a number of positively charged lysine and arginine residues along the S4 helix that permit the channel to sense voltage changes across the membrane and is responsible for channel activation [5]. In proximity to the VSD are the S5–S6 segments that form the re-entrant P-loop and constitutes the ion-selective pore of the channel [6]. Linking the four domains of Nav α subunits are multiple intracellular loops (L1–L3) in addition to cytoplasmic N- and C-termini.

**Figure 2:**
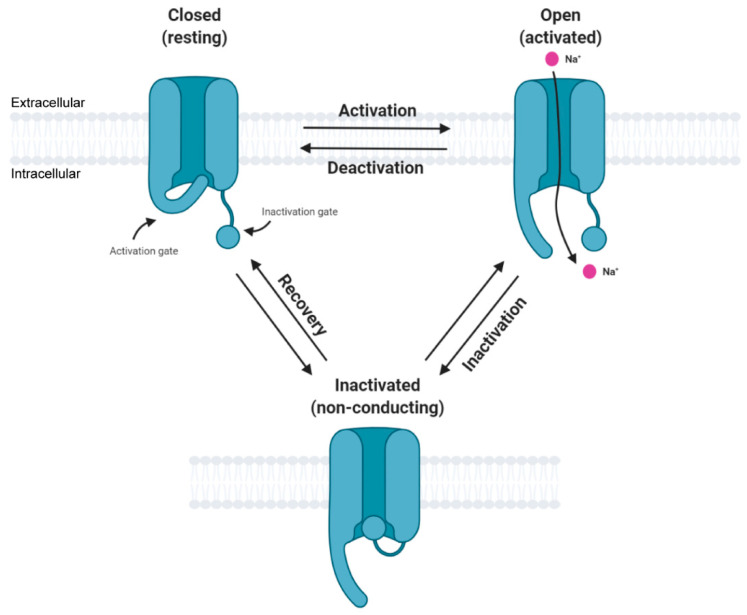
Simplified state transition model of voltage-gated sodium channels featuring closed, open, and inactivated states. This figure was created with BioRender.com.

In general, the activation cycle for Navs features transitions between resting, activated, and inactivated states (Figure 2). Under resting (hyperpolarized) conditions, Navs are in their closed state and upon depolarization transition into an open, activated state that allows for sodium ion conductance, thus initiating depolarization, and corresponds to the upstroke of the action potential. Subsequently, the channel again transitions into an inactive state, thus allowing potassium and other conductances to contribute to the downstroke of the action potential. The third intracellular loop, L3, contains an inactivation particle consisting of hydrophobic residues (isoleucine-phenylalanine-methionine, IFM motif) that is largely responsible for channel fast inactivation [7,8,9,10]. Notably, Navs can undergo various post-translational modifications (PTMs) and binding interactions with other regulatory proteins that impact their structure, function, and trafficking [11,12,13].

To date, there are nine described voltage-gated sodium channel α subunit isoforms (Na_v_1.1–Na_v_1.9) with distinct functional and pharmacological characteristics and expression patterns [14]. Sequence alignments demonstrate that the sequence homology of mammalian Nav α subunits is quite high, sharing more than 50% homology in transmembrane and extracellular domains [15]. However, Navs display greater divergence within intracellular domains. Notably, the first intracellular loop (L1) varies in length between Nav isoforms and is often the target of extensive PTMs, including phosphorylation. The intracellularly accessible regions also contain additional targets for isoform-specific regulation by other PTMs and protein-protein interactions [11,16,17,18,19].

In the 40 years since Navs were first isolated, considerable progress has been made toward mapping the vast regulatory landscape of these ion channels. However there remains much we still do not understand about Nav regulation and its impact on cellular excitability, human physiology, and disease. In the brain, the voltage-gated sodium channel Na_v_1.6 is a critical driver in the initiation and propagation of action potentials in neurons. Consequently, aberrant alterations to Na_v_1.6 activity can have profound effects on input-output properties of neurons in healthy and diverse disease states. While mutations in Na_v_1.6 may cause aberrant channel activity (i.e., channelopathies), these changes may also be the result of extensive regulation by various signaling events impacting Na_v_1.6 activity and trafficking. In this review, we will provide an overview of Na_v_1.6 in neuronal function and a comprehensive road map into the nebulous landscape of Na_v_1.6 regulation and its impact on neuronal excitability.

## 2. Na_v_1.6 Overview

### 2.1. Discovery of Na_v_1.6

The voltage-gated sodium channel isoform Na_v_1.6 is encoded by the *SCN8A* gene and is a critical driver of action potential (AP) initiation and propagation in neurons. Na_v_1.6 was identified in the mid 1990’s by two separate groups almost a decade after the first cDNA clones of Navs were isolated [3,4,20,21]. Burgess et al. [21] identified the mouse Na_v_1.6 gene using positional cloning of the mouse neurological mutant for motor end-plate disease and found this channel to be highly expressed in the brain and spinal cord, but not in skeletal muscle or heart. In parallel, Schaller and colleagues [20] detected a novel sodium channel cDNA from rat brain using RT-PCR and were the first to report the full sequence of rat Na_v_1.6. Subsequently, the gene encoding for Na_v_1.6, *SCN8A*, was mapped to chromosome 12q13 in humans [22]. Additional investigation revealed reduced sodium currents and excitability in neuronal cultures of *Scn8a* null mice and suggested that Na_v_1.6 has a powerful impact in tuning APs that underlie neuronal excitability [12,23,24,25,26,27].

### 2.2. Na_v_1.6 Expression and Distribution

Distinct from the other Nav isoforms, Na_v_1.6 is broadly expressed in the nervous system. In the central nervous system (CNS), Na_v_1.6 is prominently expressed in a variety of excitatory and inhibitory neuronal cell types, such as hippocampal pyramidal and granule cells, retinal ganglion cells, cortical pyramidal neurons, motor neurons, and cerebellar Purkinje and granule cells where it canonically contributes to electrogenesis of excitable cells [20]. Surprisingly, Na_v_1.6 is also expressed in multiple glial cells within the CNS where it has been reported to play noncanonical roles in effector functions, such as phagocytosis, migration, proliferation, and secretion of chemokines/cytokines [12,20,28]. In the peripheral nervous system (PNS), Na_v_1.6 is expressed in a variety of ganglion cells, including dorsal root ganglion and trigeminal ganglion neurons where it is critical for peripheral sensory neuron transduction [29,30,31]. Additionally, Na_v_1.6 has also been detected in Schwann cells of the PNS, however its role in Schwann cells is not well understood [20,28]. Apart from the CNS and PNS, Na_v_1.6 is also expressed at a low level in cardiomyocytes [32,33] where it is thought to function as a Ca^2+^ cycling protein within t-tubules to impact Ca^2+^ dynamics via electrogenic Na^+^-Ca^2+^ exchange [33]. Intriguingly, Na_v_1.6 also exhibits high expression in various metastatic tumors, including cancers of the breast, prostate, lymph node, and cervix, and is believed to contribute toward cancer metastasis [34,35,36].

### 2.3. Na_v_1.6 Subcellular Localization in Neurons

Neurons are highly polarized cells and their architecture is defined by two prominent subcellular compartments: (1) somatodendritic, which receive and integrate neuronal synaptic inputs, and (2) axonal, which then process and transmit these inputs to postsynaptic targets [37]. A key determinant of this neuronal polarity is the unique subcellular localization of Na_v_1.6. This channel is highly concentrated at the axon initial segment (AIS) and at nodes of Ranvier, where it plays a critical role in the initiation and propagation of APs, respectively [38,39,40,41,42,43]. The AIS is a highly specialized membrane domain about 10–60 µM in length (depending on cell type) located at the proximal end of the axon and maintains neuronal polarity by functioning as a physiological and physical bridge between somatodendritic and axonal compartments. This region is characterized by a high density of ion channels, scaffolding proteins, kinases, and other critical proteins that orchestrate AP initiation [44,45,46,47,48,49]. Specifically, Na_v_1.6 is highly concentrated in the distal half of the mature AIS, whereas Na_v_1.2 is concentrated in the proximal half [43,50].

Interestingly, the localization of Na_v_1.6 at the AIS is developmentally controlled. Studies have shown that Na_v_1.2, but not Na_v_1.6, is clustered at the developing AISs and nodes of mice up through postnatal day 10, after which a developmental switch promotes the predominant expression of Na_v_1.6 in these subcellular compartments starting in the second postnatal week and into adulthood [51,52,53]. In mature AIS, Na_v_1.6 primarily controls orthodromic AP initiation in the distal AIS down the axon, while Na_v_1.2 contributes to antidromic backpropagation of APs into the soma and dendrites [43]. Although expression of Na_v_1.6 is predominantly localized to the AIS and nodes, the channel is also expressed in somatodendritic compartments, albeit to a lesser degree. Using a highly sensitive electron microscopic immunogold technique, Lorincz and Nusser [42] determined that Na_v_1.6 expression is approximately 35–80 times higher at the AIS than at the soma or proximal and distal dendrites. Indeed, patch-clamp, sodium imaging, and similar immunogold labeling techniques in pyramidal neurons have demonstrated a sodium conductance density as high as 2500–3000 pS/µm^2^ at the AIS [42,54] versus approximately 40 pS/µm^2^ in dendrites [55].

The ability of Na_v_1.6 to localize to the AIS and axonal nodes is dependent on protein-protein interactions with AnkyrinG (AnkG); a submembranous scaffolding protein and major structural orchestrator of the AIS and nodes [56]. Specifically, studies have shown that Na_v_1.6 contains the targeting motif |(V/A)P(I/L)AXXE(S/D)D| located in the second intracellular loop (L2) that allows channels to bind AnkG and concentrate Na_v_1.6 within these axonal compartments [57,58,59,60,61]. This targeting strategy is not unique to Na_v_1.6 and also localizes Na_v_1.2, voltage-gated potassium channels, cell adhesion molecules, and other regulatory proteins to the AIS [56,62,63,64]. To this end, Nav localization to the AIS may be sensitive to post-translational modulation. A previous study has shown that casein kinase II (CK2) may phosphorylate key serine residues within the AnkG binding motif of Na_v_1.2 and regulate insertion of Na_v_1.2 at the AIS in neurons [64,65]; however, this specific regulatory tripartite protein interaction has yet to be directly identified for Na_v_1.6 channels. However, Nav localization may be governed by additional mechanisms, as the localization of Na_v_1.6 to somatodendritic compartments does not appear to rely on AnkG binding [66].

The importance of Na_v_1.6 in neuronal excitability is underscored by *Scn8a* null mice that display significantly attenuated excitatory properties due to decreased surface membrane clustering of Na_v_1.6 at the AIS and nodes [49]. Although expression of Na_v_1.6 at the AIS and nodes is crucial for the initiation and propagation of signals down the axon, its expression within dendritic compartments also impacts synaptic transmission. Nav currents have been detected in numerous hippocampal and neocortical dendrites where they function to integrate synaptic inputs and contribute to local dendritic spike generation [67,68,69,70]. Patch-clamp experiments have also demonstrated that the axonal and dendritic Nav currents differ in their biophysical properties [71,72], which might suggest different Nav isoform expression at these subcellular compartments. However, several studies have detected Na_v_1.6 as the prominent dendritic Nav at postsynaptic membranes in cerebral and cerebellar cortices [38,42,73], indicating that the same Nav isoform may dominate in adult axons and dendrites. Thus, it is likely that the activity of Na_v_1.6 at the AIS/nodes and dendrites may be differentially regulated by other mechanisms, like post-translational modifications (PTMs) and protein-protein interactions [72]. Dendritic Na_v_1.6 activity has also been shown to contribute to the generation of dendritic spikes where it is thought to promote Ca^2+^ entry in spines, essentially acting as an AP booster at the synapse [73,74,75,76,77,78] to indirectly engage Ca^2+^ signaling machinery. Thus, Na_v_1.6 appears to be the predominant Nav localized to axonal and dendritic compartments, thereby providing exquisite control over input-output properties of neurons.

### 2.4. Unique Biophysical Properties

**Figure 3:**
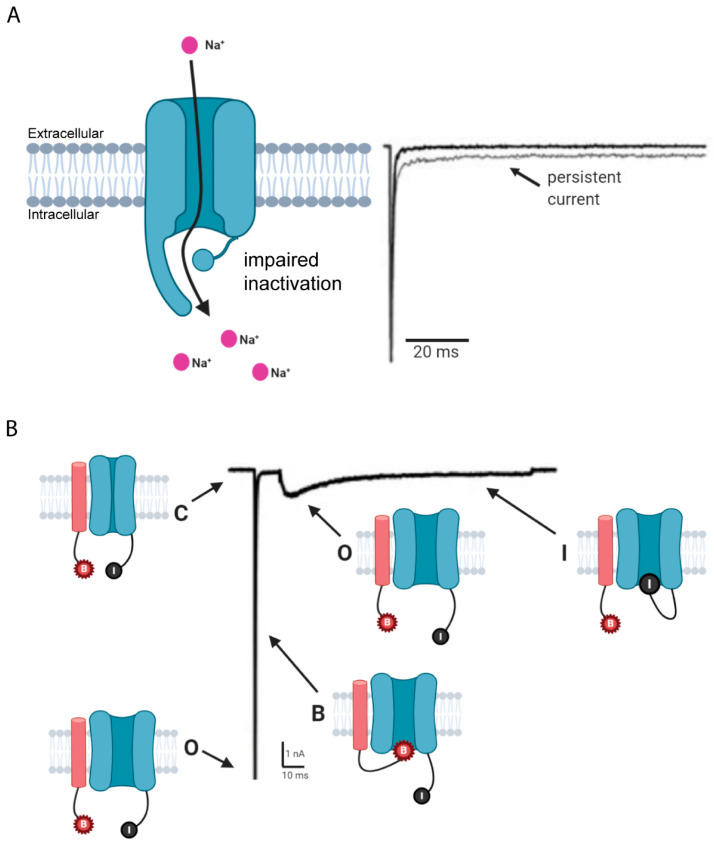
Persistent and resurgent sodium currents. (**A**) Schematic of persistent sodium current traversing the channel due to incomplete, or impaired, inactivation. (**B**) Resurgent sodium current schematic of channel conformations that have undergone open channel block (B, blocking particle; I, inactivation particle). C, closed. O, open. B, block. I, inactivated. Figure was created with BioRender.com.

Na_v_1.6 displays unique biophysical properties that enable the channel to exert powerful tuning capabilities of neuronal signals. The first functional characterizations of Na_v_1.6 α subunits in heterologous cells revealed that Na_v_1.6 currents inactivated faster than other Nav isoforms and displayed distinct sodium currents, including persistent and resurgent currents [24,79,80]. While fast-inactivating transient sodium currents are traditionally described as producing the rising phase of the AP [81], Navs can also give rise to a noncanonical subtype of non-inactivating sodium currents termed persistent sodium current [24,29,82,83] (Figure 3A). In cerebral and cerebellar neurons, persistent current is predominantly generated by Na_v_1.6 and has been reported to be approximately five-fold higher than that generated by Na_v_1.2 [84]. Although these currents are typically small (0.5–2% of peak amplitude; [85]), when summated persistent sodium currents can amplify subthreshold neuronal inputs under physiological conditions [54,77]. Consequently, persistent sodium current has been shown by modeling and electrophysiology studies to lower the threshold for AP initiation and mediate repetitive AP firing in neurons [80,83,86]. Additionally, elevated persistent currents have been shown to increase the likelihood of premature firing in neurons [87] and can undergo extensive regulation by various protein-protein interactions and PTMs [11,88,89]. The physiological importance of persistent currents is highlighted by mutational studies that either decrease or increase Na_v_1.6 persistent current generation [79,80,87,90,91,92]. For example, while cerebellar Purkinje neurons isolated from *Scn8a* null mice display a 35% decrease in the transient sodium current, they display an even larger 70% reduction in the persistent current in addition to reduced repetitive firing capabilities compared to WT littermates [80]. Conversely, transgenic mice harboring mutations that increase persistent Na_v_1.6 sodium current exhibit neuronal hyperexcitability, spontaneous seizure activity, and even sudden unexplained death [87,91,92]. Thus, persistent currents generated by Na_v_1.6 can significantly impact the initiation and propagation of APs in synaptic transmission [87,90,93,94,95].

Na_v_1.6 also displays a unique resurgent current [96]; a distinct subtype of sodium current that is a voltage- and time-dependent property of Na_v_1.6 and occurs after depolarization at intermediate repolarizing potentials to elicit a small, transient current [97] (Figure 3B). Specifically, resurgent currents occur after depolarization and channel opening in which a subset of channels can undergo a blocked state that is faster than and distinct from traditional fast inactivation. While the endogenous blocking particle may vary between neuronal subtypes, β sodium channel subunits are postulated to be key orchestrators in the generation of resurgent current [88,98,99,100,101,102]. Upon repolarization, the blocking particle unbinds, subsequently allowing for a resurgence of transient sodium current through the pore [98]. First described in cerebellar Purkinje neurons [79,80], resurgent currents are thought to contribute to spontaneous firing and multi-peaked APs. In these studies, cultures from *Scn8a* null mice displayed dramatically reduced resurgent currents and attenuated repetitive AP firing in cerebellar Purkinje neurons. Modeling and electrophysiology studies also demonstrate the importance of resurgent currents in neuronal physiology [26,91,100,101,103], revealing that aberrant resurgent current generation by Na_v_1.6 contributes to altered neuronal excitability. Together, these reports suggest that Na_v_1.6 is largely responsible for the unique sodium currents necessary for repetitive AP firing in neurons.

Apart from the channel’s distinct sodium current properties, Na_v_1.6 α subunits are also known to exhibit fast activating and fast inactivating kinetics. Additionally, Na_v_1.6 is known to display a hyperpolarized shift in the voltage-dependence of activation compared to other neuronal Navs [24,29], indicating that Na_v_1.6 is activated earlier during depolarization. As previously mentioned, Na_v_1.6 is highly concentrated at the AIS in neurons and is thought to determine firing threshold [43,50]. In cultured hippocampal neurons of *Scn8a* null mice, there is a 5 mV depolarizing shift in the voltage-dependence of activation in addition to a 60% and 75% reduction in persistent and resurgent current [49]. Furthermore, neurons isolated from these mice appear to display an 8 mV depolarizing shift in the spike threshold, making the cells less excitable. Additional studies have demonstrated that the activation threshold in the distal AIS where Na_v_1.6 concentrates is hyperpolarized by approximately 12 mV compared to the proximal AIS near the soma (−55 mV distal, −43 mV proximal [43]), consistent with a role for Na_v_1.6 in lowering the threshold for AP initiation. In total, the unique biophysical characteristics and subcellular localization of Na_v_1.6 provide flexible and complex determinants for controlling neuronal excitability.

### 2.5. Pathophysiology

As a critical driver of APs in neurons, it is no surprise that dysfunction in Na_v_1.6 may lead to aberrant neuronal activity. Mutations in Na_v_1.6 are often associated with various neuropsychiatric disorders characterized by hyperexcitability, such as pain, epilepsy, and other neurodevelopmental disorders [14,27,92,104,105,106]. The role of Na_v_1.6 in human disease was first examined in patients displaying ataxia, dystonia, tremor, and intellectual disability, phenotypes that closely resembled the defects in *Scn8a* mutant mice [21,90,107,108]. However, it was not until 2012 that the first de novo mutation (N1768D) was discovered in Na_v_1.6 using whole genome sequencing of a child with severe early-onset epileptic encephalopathy, thus directly linking channel dysfunction to pathological phenotypes [87]. Notably, Nav channel dysfunction has been increasingly linked to pathogenic changes that contribute to seizure onset in epilepsy; a debilitating neurological disorder that affects approximately 1% of the world population [109]. Over 150 distinct mutations in the *SCN8A* gene have since been identified in patients with epilepsy and account for up to 1% of epilepsies [105]. Interestingly, the majority of Na_v_1.6 mutations that have been characterized display gain-of-function effects on channel biophysical properties, including premature activation, incomplete inactivation, and increased transient, persistent, and resurgent currents; characteristics that can contribute to hyperexcitability and increased neuronal activity [27,87,91,103,104,105,110,111]. However, loss-of-function mutations in Na_v_1.6 do exist and are thought to contribute to intellectual disability [27,107].

Unfortunately, a disproportionate number of *SCN8A*-associated epilepsies remain refractory to antiepileptic treatments [27,109]. Because of high sequence homology between Nav isoforms, designing Na_v_1.6-selective drugs remains a challenge. One of the first Na_v_1.6-selective inhibitors, XEN901, has been recently reported to inhibit Na_v_1.6 by binding to the channel’s voltage sensor, thus inhibiting its recovery from inactivation [112]. While XEN901 represents a promising Na_v_1.6-selective drug, this compound has only gone through Phase I clinical trials and is still in development [113]. Interestingly, several compounds exist that have been shown to selectively target pathological currents produced by Na_v_1.6. For instance, cannabidiol and GS967 (otherwise known as Prax330) have been shown to preferentially reduce aberrant persistent and resurgent currents over transient sodium currents, however these compounds do not appear to be selective for Na_v_1.6 and can target currents in other isoforms, like Na_v_1.2 [91,114,115,116]. More recently, anti-epileptic compound screens in zebrafish models of epilepsy revealed two novel blocking compounds, MV1312 and MV1369 [117]. Although MV1312 showed a 5–6 fold selectivity of Na_v_1.6 over Na_v_1.1–Na_v_1.7, this compound displays a comparable blocking affinity for Na_v_1.8, a major PNS isoform involved in pain sensation. Similarly, while M1369 also showed higher selectivity for Na_v_1.6, this compound also blocked Na_v_1.2. Thus, identifying alternative molecular determinants, such as those involved in isoform-specific Nav modulation, may provide promising mechanisms for targeting *SCN8A*-associated pathologies.

In addition to mutations in the *SCN8A* gene, non-genetic modifications in Na_v_1.6 expression and function may also contribute to excitability disorders, such as neuropathic pain [31,118,119], autism-spectrum disorders [106,120], ischemia [121], and stress-induced disorders [122,123] in addition to epilepsy [12,27,124]. Importantly, changes in Na_v_1.6 expression have been linked to non-genetic models of acquired epilepsy, in which seizures are induced by transient brain insult or chemoconvulsants [124,125]. Following seizure onset, Na_v_1.6 expression and persistent current have been reported to increase within hippocampal regions [124,125], whereas reduction in Na_v_1.6 activity has been shown to decrease seizure susceptibility [126,127,128], suggesting an early role for Na_v_1.6 in the development of seizures. Indeed, a recent study has also demonstrated that reducing the *SCN8A* transcript by 25–50% can delay seizure onset in *SCN8A* models of epilepsy [129], indicating that a general reduction in Na_v_1.6 activity may reduce seizure susceptibility. Notably, many of the pathological changes in Na_v_1.6 function and expression are significantly influenced by various intracellular mediators including second messengers, protein-protein interactions, and PTMs. Therefore, it is critical to understand the extensive regulatory landscape contributing to Na_v_1.6 modulation and how these processes may impact neuronal excitability.

## 3. Na_v_1.6 Regulation by Protein-Protein Interactions

Sodium channels, including Na_v_1.6, are subject to extensive regulation by various auxiliary proteins and second messengers. These regulatory processes are quite powerful, displaying developmental, spatial, and temporal specificity which can be mediated by many diverse stimuli and signaling pathways. Here we will highlight several protein-protein interactions by which Na_v_1.6 is regulated and how they contribute to neuronal function.

### 3.1. Sodium Channel β Subunits

Sodium channel β subunits (β1–β4) are small single-transmembrane auxiliary proteins that can function as cell-adhesion molecules and modulate Nav surface expression and function [130]. These subunits interact with Nav α subunits non-covalently (β1 and β3) and through covalent disulfide bonds (β2 and β4) [1]. Notably, several studies have implicated β subunit regulation of Na_v_1.6 in neuronal function. Studies of β1 null mice (Scn1b^−/−^) indicate that the interaction between β1 and Na_v_1.6 is important for Na_v_1.6 function at the AIS and for neurite outgrowth [131]. Na_v_1.6-expressing cerebellar neurons of β1 null mice also display striking reductions in resurgent sodium current [131]. Moreover, the β4 subunit has also been implicated in the generation of Na_v_1.6-mediated resurgent current in Purkinje and DRG neurons [99,101,132]. These reports suggest that the C-terminal portion of β subunits may act as an open channel blocker to mediate Na_v_1.6 resurgent current. Indeed, intracellular application of a peptide mimicking this sequence, amino acids 154–167 of the β4 subunit, has been shown to recapitulate resurgent currents in heterologous expression systems lacking endogenous open channel blockers [99,100]. Interestingly, the co-expression of Nav α subunits with the full-length β4 subunit is not sufficient to produce resurgent current in heterologous expression systems [84,132], indicating that other modulatory accessory proteins, or perhaps cellular background, over-ride this function. To this end, several studies have demonstrated that various PTMs on β subunits impact β subunit interactions with Nav α subunits. For instance, phosphorylation and palmitoylation have both been implicated in β subunit regulatory properties [133,134] and suggest a complex crosstalk between Nav auxiliary proteins and PTMs on Nav α subunit function.

### 3.2. Fibroblast Growth Factor Homologous Factors

Fibroblast growth factor homologous factors (FHF1-4 also known as FGF11-14) are a family of intracellular auxiliary proteins that, contrary to their FGF counterpart, are not secreted and do not directly stimulate FGF receptors [135,136,137]. While these signaling molecules have multiple interacting partners to modulate various cellular parameters [136,137,138], FHFs can also bind to the C-terminus of Nav channel α subunits and influence both current density and gating properties [66,139,140,141,142,143]. Each member of the FHF family has at least two splice variants (A and B) with distinct N-terminal sequences [144], and their interaction with Navs produce isoform-specific changes in channel function. For example, FHF4B, which contains a unique 69 amino acid N-terminus compared to other FHFs [144,145], suppresses Na_v_1.6 sodium currents and may regulate localization of the channel to the AIS in neurons [146,147], whereas FHF4A has no effect [146]. Several studies have also shown that FHF2A and FHF2B interactions with Na_v_1.6 differentially regulate channel activity. FHF2B has been shown to increase Na_v_1.6 current density, produce a depolarizing shift in channel availability, and positively regulate resurgent currents [102,148]. In contrast, FHF2A binding to Na_v_1.6 has been shown to negatively regulate resurgent current, enhance long-term inactivation, slow the kinetics of the recovery from inactivation, and produce an even larger depolarizing shift in availability in addition to increased current density [102,146,149].

Differential modulation of Na_v_1.6 resurgent currents by FHFs has been identified as a potential mechanism underlying nociception and pain. Painful sensations often arise from increased excitability of peripheral dorsal root ganglia (DRG) neurons which are known to express Na_v_1.6-mediated resurgent currents [96]. In DRG neurons isolated from animals with radicular pain, FHF2A expression has been shown to be acutely downregulated following inflammation, whereas FHF2B expression is upregulated [102]. Notably, enhanced expression of FHF2B in pain models has been shown to contribute to increased resurgent currents in DRG neurons and mediate hyperexcitability. Interestingly, application of a peptide that mimics the FHF2A long-term inactivation particle, which negatively regulates resurgent currents, was found to reduce hyperexcitability associated with pain [102]. Importantly, these studies demonstrate that FHF-Na_v_1.6 interactions dynamically contribute to altered neuronal excitability associated with nociception and pain.

### 3.3. Ca^2+^ and Calmodulin

Intracellular Ca^2+^ is a ubiquitous second messenger critical to many aspects of neuronal function. A rapid change in the internal Ca^2+^ concentration (from 50–100 nM up to ~20 µM) is coupled to neuronal depolarization and is central to synaptic transmission [150]. Detection of this Ca^2+^ concentration change depends on Ca^2+^-binding proteins capable of translating the signal. To this end, a predominant intracellular receptor for Ca^2+^ is calmodulin (CaM), a highly conserved Ca^2+^ sensor that provides complex opportunities to functionally modulate target proteins and provide feedback for membrane excitability. The refined ability for CaM to sense Ca^2+^ is reflected in its unique structure [151,152,153,154]. This ~17 kDa protein consists of two lobes, an N-terminal (N-lobe) and C-terminal (C-lobe) lobe, and are connected by a flexible linker. Each lobe has two Ca^2+^-binding EF-hands, which can coordinate binding of one Ca^2+^ ion for a total of four Ca^2+^ ions. Interestingly, the C-lobe of CaM binds Ca^2+^ with a six-time higher affinity than the N-lobe, thereby providing CaM with the ability to sense Ca^2+^ across a dynamic concentration range [155]. Moreover, CaM undergoes a conformational change following Ca^2+^ binding that can increase or decrease the affinity of CaM to its target protein [156,157], thus allowing CaM to display a wide range of binding and regulatory properties.

Interestingly, Ca^2+^ regulation of Navs was suspected soon after the primary amino acid sequence was determined, noting that the C-terminus of Navs contained features that resembled an EF-hand Ca^2+^ binding motif [158]. Subsequent yeast two hybrid screens using the Nav CTD as bait identified CaM as a binding partner [159], leading to the identification of two CaM binding motifs in the C-terminus of Navs: (1) an “IQ” motif ([I/L/V]QXXXRGXXX[R/K]) [160] and (2) a basic amphipathic α helix, both C-terminal to the EF-hand motif. The presence of both a potential Ca^2+^ binding site and CaM binding sites in the Nav CTD suggested that Nav α subunits may be sensitive to both Ca^2+^-dependent and –independent modes of regulation. However, the ability for Ca^2+^ to directly bind the EF-hand motif of Navs and modulate channel activity remains controversial [161,162,163,164]. Studies suggest that Ca^2+^-dependent regulation of channel activity instead occurs through associated CaM [164,165] and that the structural conformation of the EF-hand motif may dictate the binding mode of CaM to the nearby IQ motif [166]. Indeed, several studies have demonstrated that CaM is able to bind to the IQ motif and modulate current density and gating properties of various Nav isoforms in an isoform-dependent manner and revealed Ca^2+^-dependent and -independent modes of Nav regulation [161,167,168,169,170,171,172,173]. Notably, Na_v_1.6 displays a higher affinity toward Ca^2+^/CaM than apo-CaM (Ca^2+^-free) binding at the channel’s IQ motif (amino acids 1902–1912; [174]), suggesting that Na_v_1.6 may be differentially modulated by CaM depending on intracellular Ca^2+^. To this end, Ca^2+^/CaM binding has been shown to delay Na_v_1.6 channel inactivation by up to 50%, whereas apo-CaM binding enhances the rate of inactivation [168]. Incidentally, the Ca^2+^/CaM-dependent slowing of inactivation kinetics could potentially prolong AP duration by enhancing neurotransmitter release at the synapse, thus contributing to increased excitability. Furthermore, apo-CaM has also been shown to differentially modulate Na_v_1.6 sodium currents, revealing reduced transient and persistent currents with decreased and increased CaM binding, respectively [89,168]. These data reveal that Navs can be dynamically modulated via Ca^2+^-dependent and -independent mechanisms. Recent studies suggest that CaM also interacts with the N-terminal domain of Na_v_1.5, suggesting that multiple CaM binding domains may shape the Nav response to Ca^2+^ signaling [175]. Whether CaM binding to the channel may be regulated by PTMs or serve as an intermediate effector between Na_v_1.6 and downstream Ca^2+^/CaM-dependent targets, like the Ca^2+^/calmodulin-dependent protein kinase II (CaMKII), remains to be determined. Intriguingly, CaM interactions with the cardiac isoform Na_v_1.5 may be influenced by CaMKII phosphorylation of the channel. Specifically, CaM binding to Na_v_1.5 has been shown to decrease following CaMKII phosphorylation at S1938 and S1989 within the CTD of the channel [176]. This suggests that the temporal order of phosphorylation events on the cardiac isoform Na_v_1.5 could potentially act as a switch to specify regulation. However, such a complex mechanism for CaMKII-dependent regulation of CaM binding to Na_v_1.6 has not yet been identified.

## 4. Post-Translational Regulation of Na_v_1.6

In addition to being regulated by various protein-protein interactions, Na_v_1.6 is also extensively modulated by post-translational modifications (PTM). PTMs are protein modifications that occur after mRNA translation into a protein and are critical for protein maturation and function. These processes can be mediated by many diverse enzymes and signaling pathways, resulting in an attachment of a biochemical group (methylation, acetylation, phosphorylation), fatty acids (palmitoylation), polypeptide (ubiquitination, SUMOylation), or more complex molecules (glycosylation) that can produce either stable or reversible changes to a protein. Importantly, PTMs display precise coupling between known interaction sites of the modifying enzyme and a given amino acid sequence on the target/substrate protein, resulting in highly specific spatial and temporal control that allows neurons to fine tune the properties of a protein, like Na_v_1.6, depending on the cellular environment and contribute to the regulation of neuronal excitability.

### 4.1. Glycosylation

A common PTM of transmembrane proteins is glycosylation, which is the attachment of glycans (carbohydrate) to a protein. Early studies indicated that glycosylation of Navs, particularly Na_v_1.2, Na_v_1.4, Na_v_1.5, Na_v_1.6, and Na_v_1.7, is a crucial step for the biosynthesis, folding, and trafficking of sodium channels [177,178,179,180,181,182,183]. Nav gating properties can also be influenced by glycosylation, altering the voltage-dependence of activation and inactivation in addition to recovery kinetics [184,185,186,187]. Mice with a single amino acid deletion within DIVS6 of Na_v_1.6 (Ile1750del) exhibit defects in glycosylation due to alterations at an adjacent glycosylation site, resulting in chronic movement disorders due to reduced channel activity and defective localization at the AIS and nodes [183]. Therefore, glycosylation is an important modification influencing the subcellular localization of Na_v_1.6 and may contribute to alterations in neuronal excitability. Future studies will be useful to determine whether similar defects in glycosylation contribute toward pathogenic mechanisms associated with patient mutations.

### 4.2. Uniquitination

Ubiquitination is a powerful PTM for modulating trafficking and cell surface expression of Navs. Mediated by ubiquitin ligases, this process refers to the covalent addition of an ubiquitin protein, a ~8.5 kDa polypeptide of 76 amino acids, to the lysine residues of a targeted protein [188]. Proteins destined for internalization through this pathway are either degraded or recycled [189,190,191], and in some instances can alter protein function. Most Navs possess a PY motif (PPXY) usually found in the C-terminus and/or L1 of channels, with the exception of Na_v_1.4 and Na_v_1.9, which allow ubiquitin ligases to bind [192,193]. Na_v_1.6 contains multiple PY motifs and undergoes ubiquitin-dependent modulation. In mouse hippocampal neurons, p38 phosphorylation of Na_v_1.6 promotes Nedd4-induced ubiquitination and internalization of the channel [122,194]. Specifically, the ubiquitin ligase Nedd4-2 has been shown to interact with two PY motifs on Na_v_1.6; the Pro-Ser-Tyr^1945^ motif in the CTD and the Pro-Gly-Ser^553^-Pro motif in L1 of the channel [194]. Both motifs were found to be necessary for Na_v_1.6 modulation by p38, which is a mitogen activated protein kinase (MAPK) implicated in relaying stress responses [194,195]. Furthermore, abrogating Nedd4-2 interactions with Na_v_1.6 was found to block channel internalization and resulted in stress-mediated increases in Na_v_1.6 currents [194]. Together, these studies highlight a complex interaction between p38 MAPK phosphorylation and ubiquitination of Na_v_1.6 and suggest that crosstalk between these different PTMs may limit neuronal excitability in response to stress-induced stimuli.

### 4.3. Palmitoylation

S-palmitoylation is a reversible PTM that involves the addition of a 16-carbon palmitic fatty acid chain to the thiol group of an intracellular cysteine of the substrate protein through thioester linkage. Palmitoylation is known to dynamically regulate diverse proteins, impacting cell surface expression, trafficking, structural conformation, protein-protein interactions, and function [178,196,197]. Palmitoylation also plays crucial roles in ion channel regulation and is involved in various phases of the ion channel life cycle, including synthesis, maturation, trafficking, subcellular localization, and internalization [196]. The first characterization of S-palmitoylation of voltage-gated sodium channels identified this process to regulate the early stages of protein biosynthesis [178]. Recently, Na_v_1.6 was identified as a novel target for regulation by S-palmitoylation [198]. This study identified two palmitoylation sites (C1169, C1170) in L2 of the channel that appear to be responsible for modulating voltage-dependence of inactivation, and one site in the C-terminus (C1978) exclusive to Na_v_1.6 that enhances Na_v_1.6 current density [198]. Further characterization of these sites revealed a novel role of Na_v_1.6 palmitoylation in regulating neuronal excitability [198], showing that the ablation of C1169, C1170, and C1978 results in a substantial reduction in Na_v_1.6-mediated excitability of DRG neurons, indicating that targeting Na_v_1.6 palmitoylation may represent a potentially useful strategy to reduce neuronal excitability.

### 4.4. Phosphorylation

Phosphorylation is a crucial PTM that affects up to 30% of proteins in cells at any given time [199]. Catalyzed by protein kinases, this PTM is characterized by the reversible covalent addition of a negatively charged (−2) phosphate group onto a serine, threonine, or tyrosine residue of a target protein:

MgATP^1−^ + protein–O:H → protein–O:PO_3_^2−^ + MgADP + H^+^.

Phosphorylation is perhaps the most extensively studied Nav PTM and has been shown to target multiple regions of sodium channels [11,16,17,18,19]. Nav phosphorylation is carried out by diverse kinases that can modulate various aspects of channel function. This kinase diversity represents multiple signaling pathways that enable Nav modulation in concert with other pathways, or distinctively by different second messengers, thus providing a trove of potential regulation of neuronal activity. For example, sodium channels from the CNS (Na_v_1.1 and Na_v_1.2), PNS (Na_v_1.7 and Na_v_1.8), cardiac tissue (Na_v_1.5), and skeletal muscle (Na_v_1.4) are modulated by the cAMP-dependent protein kinase PKA and/or PKC, which can be activated by Ca^2+^/lipid hydrolysis, producing differential effects on channel activity [13,200]. While PKC appears to consistently attenuate sodium currents across most isoforms [201,202,203,204,205,206], the effects of PKA phosphorylation are more diverse, resulting in attenuated tetrodotoxin-sensitive (TTX-S) sodium currents [202,207,208,209] while potentiating TTX-resistant (TTX-R) sodium currents [202,210,211,212], and producing shifts in voltage-dependent gating properties. The PKA phospho-sites S573 and S687, and the PKC phospho-site S576, for example, have been shown to contribute to the functional modulation of Na_v_1.2 sodium currents [206,208,213,214]. Interestingly, despite carrying homologous PKA and PKC phospho-sites, Na_v_1.6 appears to be largely resistant to modulation by these kinases in neurons [84], suggesting that Na_v_1.6 modulation may be targeted through a different signaling pathway.

**Figure 4:**
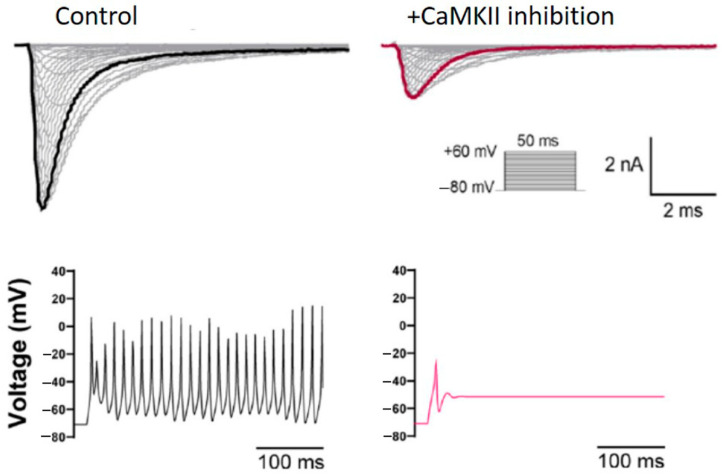
CaMKII modulates Na_v_1.6 activity and neuronal excitability. CaMKII inhibition reduces Na_v_1.6 sodium currents (**top**) and neuronal excitability (**bottom**) in simulated Purkinje neurons. This research was originally published in the Journal of Biological Chemistry [11], © the American Society for Biochemistry and Molecular Biology.

**Figure 5:**
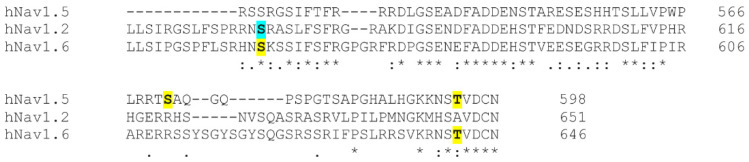
Sequence alignment spanning homologous phosphorylation sites in Na_v_1.2, Na_v_1.5, and Na_v_1.6 in the L1 region between domains I and II. Blue represents PKA phosphorylation site. Yellow represents CaMKII phosphorylation site.

To this end, Na_v_1.6 has been recently identified as a target for modulation by CaMKII (Figure 4) [11]. CaMKII is a multifunctional Ser/Thr protein kinase highly concentrated in the brain and is implicated in the physiological and pathophysiological regulation of excitability [215]. Acute CaMKII inhibition has been shown to produce loss-of-function effects in Na_v_1.6 activity, including decreased transient and persistent Na_v_1.6 sodium currents in Purkinje neurons in addition to a depolarized shift in the voltage-dependence of activation in cells heterologously expressing Na_v_1.6. Further modeling the effects of CaMKII inhibition on Na_v_1.6 activity in Purkinje neurons has shown significantly reduced spontaneous and evoked excitability, suggesting that this mechanism may be important in regulating neuronal function [11]. Importantly, CaMKII modulation of Na_v_1.6 is mediated by phosphorylation of the channel at two distinct sites in the L1 region, including S561 and T642. This is consistent with previous reports identifying L1 as a hotspot for Nav PTMs and regulation [16,18,19,216,217,218]. Notably, the CaMKII-dependent phosphorylation sites S561 and T642 in Na_v_1.6 display homologous sites of regulation in other Nav isoforms (Figure 5). To date, Na_v_1.6 appears largely resistant to modulation by PKA [84]. While phosphorylation of S573 in Na_v_1.2 has been shown to mediate PKA-dependent reductions in Na_v_1.2 sodium currents [208], phosphorylation of S561 in Na_v_1.6 has been implicated in CaMKII-dependent modulation of the voltage-dependence of activation [11]. Moreover, CaMKII phosphorylation of Na_v_1.6 at T642 has been implicated in sodium current regulation, while CaMKII phosphorylation at the equivalent T594 site in Na_v_1.5 has been shown to regulate channel gating properties [11,18]. Together, these studies stress the intricacies underlying isoform-selectivity of CaMKII modulation and further highlight the diverse functional responses to phosphorylation of Navs at homologous sites by the same kinase or distinct signaling pathways. The possibility for CaMKII-dependent modulation of Na_v_1.6 is a fascinating nexus between a kinase implicated in synaptic plasticity and a channel critical for the initiation and propagation of APs. Additional studies investigating this relationship will be important to determine how this mechanism regulates neuronal excitability in physiology and disease.

As discussed above, Na_v_1.6 is also modulated by p38 mitogen-activated protein kinase (MAPK). This kinase is classically linked to environmental stressors, including cell injury and hypoxia. Several TTX-S (Na_v_1.6 and Na_v_1.7) and TTX-R (Na_v_1.8 and Na_v_1.9) Navs can be subject to phosphorylation by these pathways and modulate aspects of their function and surface expression [119]. Phosphorylation of Na_v_1.6 by activated p38 occurs within L1, specifically at S553, which results in a reduction of Na_v_1.6 current [122]. As previously mentioned, p38 phosphorylation of Na_v_1.6 promotes Nedd4-induced ubiquitination of the channel to reduce Na_v_1.6 sodium current [194]. Two other major kinases included in the MAPK family are c-Jun N-terminal kinases (JNKs) and extracellular signal-regulated kinases (ERKs). Direct modulation of Na_v_1.6 by either of these kinases has yet to be identified; however, indirect modulation of Na_v_1.6 by JNK has been observed and is thought to contribute to Alzheimer’s disease (AD) pathogenesis [219]. In models of AD, the amyloid precursor protein (APP) has been shown to upregulate Na_v_1.6 expression and activity, which may contribute to membrane depolarization and increased spike frequency, thereby resulting in neuronal hyperexcitability [219,220,221,222]. The reciprocal has also been shown, whereby APP knockdown can reduce Na_v_1.6 expression and activity [222]. Interestingly, the ability of APP to modulate Na_v_1.6 sodium currents is mediated by activation of JNK, which in turn enables APP to upregulate Na_v_1.6 cell surface expression and enhance sodium current [219]. Together, these studies indicate that Na_v_1.6 modulation through MAPK pathways is complex and may be a critical player in pathophysiological neuronal excitability.

Several studies have also identified a role for glycogen synthase kinase-3 (GSK3) in regulating Na_v_1.6 activity. Beyond regulation of glycogen metabolism, this kinase plays important roles in the regulation of neuronal development and function, including synaptic plasticity and neuronal excitability [223,224,225]. A previous report demonstrated that pharmacological inhibition and genetic silencing of GSK3β produces loss-of-function effects on channel activity, resulting in decreased transient and persistent Na_v_1.6 sodium currents in addition to a leftward shift in channel availability [226]. In this work it was shown that GSK3β phosphorylates T1936 in the Na_v_1.6 CTD and that the interaction is important in regulating excitability of medium spiny neurons in the nucleus accumbens, implicating this mechanism in the dopamine reward pathway. A recent study suggests that FHF4 binding with the Na_v_1.6 CTD may be regulated by GSK3β phosphorylation of either FHF4, Na_v_1.6, or potentially both [227,228]. In particular, inhibiting GSK3β was found to decrease FHF4:Na_v_1.6 complex formation, which subsequently suppressed neuronal excitability and suggests that multiplexed signaling pathways are major determinants underlying Na_v_1.6 regulation and neuronal function [228,229,230].

## 5. Conclusions

Significant progress has been made toward understanding the intricate regulation of Na_v_1.6 in neuronal function, however the picture is far from complete. Navs undergo remarkably complex and extensive modes of regulation by many different auxiliary proteins and post-translational mechanisms, each of which are subject to regulation themselves by diverse signaling pathways. Although this review examined several aspects of Na_v_1.6 regulation, it is likely that Na_v_1.6 is sensitive to additional protein-protein interactions and PTMs that have yet to be identified. Furthermore, considerable crosstalk occurs between different modes of regulation, making it difficult to predict how a particular ensemble of modifications may impact channel properties and neuronal excitability. Overall, the studies reviewed here expand our current knowledge of Na_v_1.6 regulation and highlight important modulatory mechanisms mediating changes in neuronal excitability associated with health and disease.
